# Incidence of Dementia Following Hospitalization With Infection Among Adults in the Atherosclerosis Risk in Communities (ARIC) Study Cohort

**DOI:** 10.1001/jamanetworkopen.2022.50126

**Published:** 2023-01-09

**Authors:** Bruno Bohn, Pamela L. Lutsey, Jeffrey R. Misialek, Keenan A. Walker, Charles H. Brown, Timothy M. Hughes, Junichi Ishigami, Kunihiro Matsushita, Ryan T. Demmer

**Affiliations:** 1 Division of Epidemiology and Community Health, University of Minnesota School of Public Health, Minneapolis; 2 Laboratory of Behavioral Neuroscience, National Institute on Aging, National Institutes of Health, Baltimore, Maryland; 3 Department of Anesthesiology and Critical Care Medicine, Johns Hopkins University School of Medicine, Baltimore, Maryland; 4 Department of Internal Medicine, Wake Forest School of Medicine, Winston-Salem, North Carolina; 5 Department of Epidemiology, Johns Hopkins Bloomberg School of Public Health, Baltimore, Maryland; 6 Department of Epidemiology, Columbia University Mailman School of Public Health, New York, New York

## Abstract

**Importance:**

Factors associated with the risk of dementia remain to be fully understood. Systemic infections are hypothesized to be such factors and may be targets for prevention and screening.

**Objective:**

To investigate the association between hospitalization with infection and incident dementia.

**Design, Setting, and Participants:**

Data from the community-based Atherosclerosis Risk in Communities (ARIC) study, a prospective cohort study, were used. Enrollment occurred at 4 research centers in the US, initiated in 1987 to 1989. The present study includes data up to 2019, for 32 years of follow-up. Data analysis was performed from April 2021 to June 2022.

**Exposures:**

Hospitalizations with infections were identified via medical record review for selected *International Classification of Diseases, Ninth Revision* (*ICD-9*) and *International Statistical Classification of Diseases and Related Health Problems, Tenth Revision* (*ICD-10*) codes, from baseline until administrative censoring or dementia diagnosis. Participants were considered unexposed until first hospitalization with infection and exposed thereafter. Selected infection subtypes were also considered.

**Main Outcomes and Measures:**

Incident dementia and time-to-event data were identified through surveillance of *ICD-9 *and *ICD-10* hospitalization and death certificate codes, in-person assessments, and telephone interviews. A sensitivity analysis was conducted excluding cases occurring within 3 years or beyond 20 years from exposure. Data were collected before study hypothesis formulation.

**Results:**

Of the 15 792 ARIC study participants, an analytical cohort of 15 688 participants who were dementia free at baseline and of Black or White race were selected (8658 female [55.2%]; 4210 Black [26.8%]; mean [SD] baseline age, 54.7 [5.8] years). Hospitalization with infection occurred among 5999 participants (38.2%). Dementia was ascertained in 2975 participants (19.0%), at a median (IQR) of 25.1 (22.2-29.1) years after baseline. Dementia rates were 23.6 events per 1000 person-years (95% CI, 22.3-25.0 events per 1000 person-years) among the exposed and 5.7 events per 1000 person-years (95% CI, 5.4-6.0 events per 1000 person-years) among the unexposed. Patients hospitalized with infection were 2.02 (95% CI, 1.88-2.18; *P* < .001) and 1.70 (95% CI, 1.55-1.86; *P* < .001) times more likely to experience incident dementia according to unadjusted and fully adjusted Cox proportional hazards models compared with individuals who were unexposed. When excluding individuals who developed dementia less than 3 years or more than 20 years from baseline or the infection event, the adjusted hazard ratio was 5.77 (95% CI, 4.92-6.76; *P* < .001). Rates of dementia were significantly higher among those hospitalized with respiratory, urinary tract, skin, blood and circulatory system, or hospital acquired infections. Multiplicative and additive interactions were observed by age and *APOE-ε* genotype.

**Conclusions and Relevance:**

Higher rates of dementia were observed among participants who experienced hospitalization with infection. These findings support the hypothesis that infections are factors associated with higher risk of dementias.

## Key Points

**Question:**

Are hospitalizations with infection associated with dementia incidence?

**Findings:**

This cohort study included 15 688 participants over 32 years of follow-up in the Atherosclerosis Risk in Communities study. Participants who were hospitalized with infection were 1.7 times more likely to experience incident dementia compared with those who were unexposed.

**Meaning:**

These findings suggest that infections are associated with incident dementias, and their prevention could be important for dementia prevention.

## Teaser

This cohort study examines the association between hospitalization with infection and incident dementia among participants in the Atherosclerosis Risk in Communities (ARIC) study.

## Introduction

Dementias currently affect an estimated 50 million people worldwide, and 152 million prevalent cases are projected by 2050.^1,2^ Dementia describes symptoms affecting memory, cognitive ability, and behavior, with Alzheimer disease (AD) being its most common cause.^3^ Factors associated with the risk of dementia remain poorly understood, with only one-half of AD cases being attributable to known modifiable factors.^4^

Among many theorized mechanisms contributing to dementia causes, neuroinflammation has been recognized as a likely factor.^3^ In the case of AD, the most studied form of dementia, neuroinflammation is hypothesized to affect disease onset and progression.^3^ Several pathogenic mechanisms in the central nervous system, including astrogliosis and microgliosis, have been hypothesized to underly AD causes and progression.^5^ The connection between these pathogenic mechanisms, neuroinflammation, and cognitive decline has been shown in murine models.^6^

Although neuroinflammation may occur as a result of central nervous system immune cell overactivity, recent evidence suggests that the peripheral immune system may also be relevant.^5,7^ Higher levels of proinflammatory cytokines have been reported among patients with AD and other neurodegenerative diseases, with associations suggested from prospective studies linking inflammation to adverse neurocognitive outcomes.^7^ Growing evidence links neuroinflammation to systemic or vascular inflammation from peripheral infections through both humoral and neural pathways.^7^ Furthermore, neuroinflammation may remain long after resolution of a peripheral infection, highlighting the potential impact infections can have on chronic disease development, including dementia.^7^ Thus, systemic infections are hypothesized to activate the brain’s immune system and exacerbate or initiate neuroinflammation, thereby increasing dementia risk.

The association between infection and neurodegenerative diseases has been reported previously.^8^ Brown et al^9^ showed that hospitalization was associated with cognitive decline over a 11-year period, although infections resulting in hospitalization specifically were not investigated. Walker et al^10^ used data from the Atherosclerosis Risk In Communities (ARIC) study over a 24-year follow-up period to show that hospitalized participants were more likely to have greater white matter hyperintensity volume and lower white matter microstructural integrity. Similarly, recent UK Biobank findings suggest that SARS-CoV-2 infection might be associated with brain abnormalities and cognitive decline.^11^ Sipilä et al^12^ found that infections resulting in hospitalization were associated with 60% higher dementia risk in 3 racially homogenous Finnish cohorts (median follow-up of 19 years).

The present study includes a large, racially diverse cohort with comprehensive demographic, behavioral, clinical, and *APOE*-ε4 genotype information and stringent ascertainment of dementia over a 32-year follow-up in the ARIC study. In addition, we build on prior literature by examining whether systemic infections are better estimates of incident dementia than localized infections (eg, gastrointestinal infections). We hypothesize greater risk of incident dementia among participants experiencing hospitalization with infection (HWI) compared with participants who did not experience HWI over the course of follow-up.

## Methods

### Study Population

The ARIC study is a community-based prospective cohort study in the US.^13^ Enrollment of 15 792 participants occurred from 1987 to 1989 in suburbs of Minneapolis, Minnesota; Washington County, Maryland; Forsyth County, North Carolina; and Jackson, Mississippi. The study was reviewed and approved by institutional review boards at each study center. All participants provided written informed consent. The present analysis includes follow-up through December 2017 for the Jackson center and December 2019 for other centers. This study follows the Strengthening the Reporting of Observational Studies in Epidemiology (STROBE) reporting guidelines for cohort studies.

Because of the low numbers, Black participants from Minnesota and Maryland and those reporting race other than White or Black were removed, a standard approach in ARIC analyses. Participants with dementia diagnosis at baseline were excluded.

### Hospitalization With Infection

Hospitalizations were identified through telephone calls, local hospitals surveillance, and death interviews with proxies. The main exposure was first occurrence of HWI, identified by *International Classification of Diseases, Ninth Revision (ICD-9)* or *International Statistical Classification of Diseases and Related Health Problems, Tenth Revision (ICD-10)* codes (eTable 1 in Supplement 1) in the first 5 *ICD* positions, as previously performed in ARIC.^14^ HWI was treated as time-varying, with participants considered unexposed until first HWI and exposed thereafter. For primary analyses, the first occurrence of any HWI was considered. Secondary analyses considered respiratory, urinary tract, digestive tract, skin, blood or circulatory system, and hospital-acquired infections, separately. Sensitivity analyses were conducted by defining HWI as a primary diagnosis (*ICD* position 1).

### Assessment of Dementia

Methods for dementia ascertainment in ARIC have been described previously.^15^ Dementia cases were identified through surveillance of *ICD* and death certificate codes, in-person assessments, and telephone interviews.^15^ Detailed dementia information was collected starting in 2011 to 2013, with regular telephone calls (annually before 2012 and twice yearly thereafter). In-person cognitive testing took place in the fifth and sixth (2016-2017) visits of the ARIC–Neurocognitive Study, accounting for results from previous cognitive assessments, informant interviews, and a complete neurophysiological examination.^15^ For those who did not attend ARIC–Neurocognitive Study clinic visits, Telephone Instrument of Cognitive Status–modified or a telephone interview with an informant were used.^15^ The Six-Item Screener was also used after 2013 to assess between-visit cognition.

A sensitivity analysis was conducted by censoring dementia cases occurring less than 3 years or more than 20 years after a HWI to remove cases that are less plausibly linked to infection events or instances in which undiagnosed dementia or other cognitive decline could increase infection risk. For those who did not have a HWI event, dementias occurring less than 3 years or more than 20 years after baseline were censored as event free to prevent bias.

### Risk Factor Measurements

Participant demographic, behavioral, *APOE*-ε4 genotype, and clinical characteristics were collected at baseline and subsequent visits. Information on participant age, sex, race, education level, smoking habits, and income was collected via questionnaire at baseline. Race was used in the study as a proxy for race-based discrimination, structural and systemic racial inequities, and their effects on socioeconomic factors, health, and wellness. Baseline blood was collected following an 8-hour fast for measurement of glucose, high-density lipoprotein cholesterol, low-density lipoprotein cholesterol (estimated via the Friedewald equation^16^), and triglycerides. *APOE*-ε4 genotyping was performed using the TaqMan assay (Applied Biosystems), with participants categorized as carriers (1 or 2 alleles) or noncarriers (0 alleles). Body mass index (calculated as weight in kilograms divided by height in meters squared) was derived from baseline weight and height. Blood pressure was measured at baseline, in triplicate, following a 5-minute rest, with the average between second and third measurements being used for analysis. Baseline antihypertensive medication use was determined via self-report or through drug codes from participant-brought medications. Diabetes was defined through self-reported diagnosis, fasting glucose greater than or equal to 126 mg/dL (to convert to millimoles per liter, multiply by 0.555), nonfasting glucose greater than or equal to 200 mg/dL, or pharmacological treatment for diabetes. Baseline prevalence of heart failure, coronary heart disease, myocardial infarction, atrial fibrillation, and stroke, including transient ischemic attack (TIA), were defined via self-report.

### Statistical Analysis

Data analysis was performed from April 2021 to June 2022. Analyses were conducted using R statistical software version 4.1.3 (R Project for Statistical Computing). Participant characteristics were summarized across outcome and exposure groups through percentages, means (SDs), or medians (IQRs). Time-varying HWI was the primary exposure, and time to dementia diagnosis was the primary outcome. Follow-up time began at baseline (1987-1989) and accrued until date of dementia diagnosis, loss to follow-up, death, or administrative censoring (December 31, 2019). Hazard ratios (HRs) and 95% CIs were calculated using multivariable Cox proportional hazards models. Four multivariable models were used with sequential addition of demographic variables (model 1), behavioral variables (model 2), risk biomarkers (model 3), and comorbidities (model 4), in an effort to minimize collinearity and preserve parsimony. The 95% CIs for incidence rate differences were computed with the Poisson distribution.^17^ Participants with missing data on covariates were excluded from adjusted analyses. Two-sided *P* < .05, calculated with *t*, χ^2^, Fisher exact, or Wilcoxon rank-sum tests, as appropriate, was considered significant.

The association between HWI (*ICD* positions 1-5) and incident dementia was also investigated in subgroups defined by median age at baseline, *APOE*-ε4 genotype, prevalent stroke or TIA, or sex. Statistical interaction on the multiplicative scale was investigated through interaction terms for each of these variables and HWI. Statistical interaction on the additive scale was summarized with incidence rate differences for each strata, and statistical significance of differences between strata was assessed with the test for homogeneity of incidence rate differences.^17^

## Results

### Cohort Description

The present study included 15 688 participants of the ARIC study, of whom 8658 (55.2%) were female and 4210 (26.8%) were Black, with a mean (SD) age at baseline of 54.7 (5.8) years. Characteristics of this cohort by dementia diagnosis are described in Table 1. Participants who developed dementia were more likely to be older, female, Black, or *APOE*-ε4 carriers. Several baseline vascular factors, including high blood pressure and previous stroke or TIA, were also associated with higher dementia incidence. Participant characteristics according to HWI (*ICD* positions 1-5) are summarized in eTable 2 in Supplement 1. There was substantial overlap between the aforementioned factors for incident dementia and for incident HWI; older age, current or former smoking, lower education, *APOE*-ε4 noncarrier status, and several vascular factors were associated with HWI.

**Table 1.** Cohort Characteristics by Incident Dementia Diagnosis Among Participants in the Atherosclerosis Risk in Communities Study (1987-2019)

Characteristic	Patients, No. (%)			*P* value
	All (N = 15 688)	No dementia (n = 12 713)	Incident dementia (n = 2975)	
Time to dementia diagnosis, median (IQR), y	NA	NA	25.1 (22.2-29.1)	NA
Demographics				
Age at baseline, mean (SD), y	54.7 (5.8)	54.1 (5.7)	56.9 (5.4)	<.001^a^
Age at or above the median (≥54.45 y)	7844 (50.0)	5871 (46.2)	1973 (66.4)	<.001^b^
Sex				
Female	8658 (55.2)	6907 (54.3)	1751 (58.9)	<.001^b^
Male	7030 (44.8)	5806 (45.7)	1224 (41.1)	
Race				
Black	4210 (26.8)	3300 (26.0)	910 (30.6)	<.001^b^
White	11478 (73.2)	9413 (74.0)	2065 (69.4)	
Race by center				
Black, Mississippi	3728 (23.8)	2889 (22.7)	839 (28.2)	<.001^b^
Black, North Carolina	482 (3.1)	411 (3.2)	71 (2.4)	
White, Maryland	3975 (25.3)	3138 (24.7)	837 (28.1)	
White, Minnesota	3972 (25.3)	3308 (26.0)	664 (22.3)	
White, North Carolina	3531 (22.5)	2967 (23.3)	564 (19.0)	
Behaviors				
Ever drinker (missing = 80)	11683 (74.9)	9664 (76.4)	2019 (68.2)	<.001^b^
Ever smoker (missing = 11)	9152 (58.4)	7593 (59.8)	1559 (52.4)	<.001^b^
Education (missing = 26)				
Less than high school	3736 (23.9)	2834 (22.3)	902 (30.3)	<.001^b^
High school degree (or equivalent)	6380 (40.7)	5214 (41.1)	1166 (39.2)	
More than high school	5546 (35.4)	4642 (36.6)	904 (30.4)	
*APOE*-ε4 genotype (missing = 679)				
Negative	10379 (69.2)	8728 (71.8)	1651 (58.0)	<.001^b^
Positive	4630 (30.8)	3434 (28.2)	1196 (42.0)	
Biomarkers at baseline				
Low-density lipoprotein cholesterol, mean (SD), mg/dL (missing = 476)	137.7 (39.4)	136.5 (39.0)	142.7 (40.4)	<.001^a^
High-density lipoprotein cholesterol, mean (SD), mg/dL (missing = 249)	51.6 (17.1)	51.5 (17.2)	52.1 (16.9)	.07^a^
Triglycerides, median (IQR), mg/dL (missing = 248)	110 (79-157)	109 (78-156)	115 (81-164)	<.001^c^
Estimated glomerular filtration rate serum creatinine, mean (SD), mL/min/1.73 m^2^ (missing = 149)	102.4 (15.9)	102.7 (16.0)	101.5 (15.2)	<.001^b^
Comorbidities at baseline				
High blood pressure (missing = 80)	5471 (35.1)	4289 (33.9)	1182 (39.9)	<.001^b^
Diabetes (missing = 147)	1863 (12.0)	1492 (11.8)	371 (12.6)	.29^b^
Atrial fibrillation (missing = 224)	37 (0.2)	27 (0.2)	10 (0.3)	.29^b^
Stroke or transient ischemic attack (missing = 3629)	732 (6.1)	582 (5.9)	150 (6.7)	.17^b^
Heart failure (missing = 282)	746 (4.8)	616 (4.9)	130 (4.5)	.29^b^
Myocardial infarction (missing = 231)	650 (4.2)	568 (4.5)	82 (2.8)	<.001^b^
Coronary heart disease (missing = 339)	763 (5.0)	663 (5.3)	100 (3.5)	<.001^b^

Abbreviation: NA, not applicable.

SI conversion factors: To convert high-density lipoprotein cholesterol to millimoles per liter, multiply by 0.0259; low-density lipoprotein cholesterol to millimoles per liter, multiply by 0.0259; triglycerides to millimoles per liter, multiply by 0.0259.

^a^ *P* value was calculated with *t* test.

^b^ *P* value was calculated with χ^2^ or Fisher exact test, when appropriate.

^c^ *P* value was calculated with Wilcoxon rank test.

**Figure 1.**
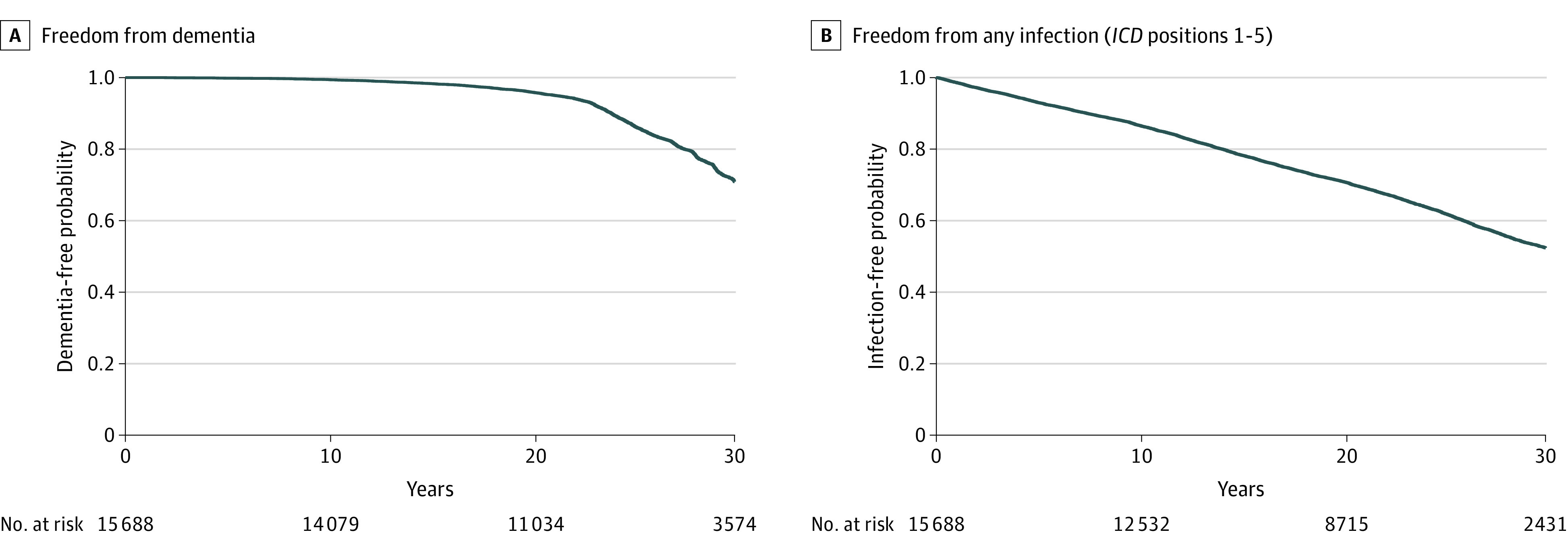
Survival Plots of Incident Dementia and Any Incident Infection With Hospitalization, the Atherosclerosis Risk in Communities Study, 1987-2019 In both panels, participants censored at loss to follow-up, death, or incident dementia. In panel B only (*International Classification of Diseases, Ninth Revision *or* Tenth Revision* positions 1-5), participants also were censored at infection with hospitalization occurrence.

During a maximum follow-up of 32 years, 2975 participants (19.0%) received a diagnosis of dementia, with a median (IQR) time to diagnosis of 25.1 (22.2-29.1) years. The cohort contributed to a total of 361 331 person-years, resulting in an incidence rate of dementia of 8.2 events per 1000 person-years (95% CI, 7.9-8.5 events per 1000 person-years) (Figure 1A).

The predementia diagnosis cumulative incidence of any HWI was 38.2% (5999 patients). Cumulative incidences of infection subtypes are described in eTable 3 in Supplement 1. Respiratory and urinary tract infections were the most common, with cumulative incidences of 19.7% and 12.4%, respectively. Freedom from any infection (predementia) is depicted in Figure 1B.

### Any Hospitalization With Infection and Dementia

The rate of dementia was 23.6 events per 1000 exposed person-years (95% CI, 22.3-25.0 events per 1000 exposed person-years), in contrast to a rate of 5.7 events per 1000 unexposed person-years (95% CI, 5.4-6.0 events per 1000 unexposed person-years). In an unadjusted model (model 0), those who had any HWI had approximately 2 times higher risk of incident dementia than those without HWI (HR, 2.02; 95% CI, 1.88-2.18; *P* < .001). HWI remained significantly associated with dementia diagnosis after full adjustments, with a 70% increase in risk (Table 2) (HR, 1.70; 95% CI, 1.55-1.86; *P* < .001). When restricting exposure to HWI as primary diagnosis (*ICD* position 1), the rate of dementia among the exposed (26.7 events per 1000 person-years; 95% CI, 24.9-28.7 events per 1000 person-years) remained higher than among the unexposed (6.7 events per 1000 person-years; 95% CI, 6.4-7.0 events per 1000 person-years), as summarized in eTable 4 in Supplement 1. As in the primary analysis, participants with any HWI had a 65% higher risk of dementia diagnosis (fully adjusted model, HR, 1.65; 95% CI, 1.49-1.83; *P* < .001).

**Table 2.** Multivariable Adjusted Association Between Any Hospitalization With Infection and Infection Subtypes (*International Classification of Diseases, Ninth Revision *or* Tenth Revision* Positions 1-5) and Incident Dementia Among Participants in the Atherosclerosis Risk in Communities Study (1987-2019)

Exposure construct and infection status	Patients, No. (N = 15 688)		Rate, No. of cases per 1000 person-years (95% CI)	HR (95% CI)^a^				
	Total	With dementia		Model 0	Model 1	Model 2	Model 3	Model 4
Any infection								
Yes	5999	1210	23.6 (22.3-25)	2.02 (1.88-2.18)^b^	1.69 (1.57-1.83)^b^	1.69 (1.57-1.82)^b^	1.63 (1.51-1.76)^b^	1.7 (1.55-1.86)^b^
No	9689	1765	5.7 (5.4-6)	1 [Reference]	1 [Reference]	1 [Reference]	1 [Reference]	1 [Reference]
Respiratory infection								
Yes	3083	566	26.9 (24.8-29.1)	1.93 (1.76-2.12)^b^	1.56 (1.42-1.71)^b^	1.55 (1.42-1.7)^b^	1.51 (1.37-1.66)^b^	1.55 (1.38-1.73)^b^
No	12 605	2409	7.1 (6.8-7.4)	1 [Reference]	1 [Reference]	1 [Reference]	1 [Reference]	1 [Reference]
Urinary tract infection								
Yes	1943	473	38.7 (35.4-42.3)	2.48 (2.24-2.74)^b^	1.94 (1.75-2.14)^b^	1.94 (1.76-2.15)^b^	1.86 (1.67-2.07)^b^	1.98 (1.76-2.24)^b^
No	13 745	2502	7.2 (6.9-7.5)	1 [Reference]	1 [Reference]	1 [Reference]	1 [Reference]	1 [Reference]
Digestive tract infection								
Yes	581	121	20.2 (16.9-24.1)	1.26 (1.05-1.51)^c^	1.24 (1.03-1.49)^c^	1.22 (1.02-1.47)^c^	1.13 (0.93-1.37)	1.03 (0.81-1.3)
No	15 107	2854	8 (7.7-8.3)	1 [Reference]	1 [Reference]	1 [Reference]	1 [Reference]	1 [Reference]
Skin infection								
Yes	919	198	30.3 (26.4-34.8)	1.96 (1.69-2.26)^b^	1.69 (1.46-1.95)^b^	1.69 (1.46-1.95)^b^	1.65 (1.42-1.92)^b^	1.67 (1.4-2)^b^
No	14 769	2777	7.8 (7.5-8.1)	1 [Reference]	1 [Reference]	1 [Reference]	1 [Reference]	1 [Reference]
Blood or circulatory system infection								
Yes	191	40	38.9 (28.7-52.7)	2.22 (1.62-3.03)^b^	1.93 (1.41-2.64)^b^	1.95 (1.43-2.67)^b^	1.91 (1.38-2.64)^b^	2.13 (1.45-3.12)^b^
No	15 497	2935	8.1 (7.9-8.4)	1 [Reference]	1 [Reference]	1 [Reference]	1 [Reference]	1 [Reference]
Hospital-acquired infection								
Yes	499	97	27.7 (22.8-33.8)	1.96 (1.6-2.41)^b^	1.72 (1.41-2.11)^b^	1.7 (1.39-2.09)^b^	1.73 (1.39-2.14)^b^	1.96 (1.52-2.51)^b^
No	15 189	2878	8 (7.8-8.3)	1 [Reference]	1 [Reference]	1 [Reference]	1 [Reference]	1 [Reference]

Abbreviation: HR, hazard ratio.

^a^ HRs were derived from Cox proportional hazards models. Model 0 was unadjusted (15 688 patients at risk). Model 1 was adjusted for age, sex, race by center, and education (15 662 patients at risk). Model 2 included model 1 plus smoking and drinking (15 581 patients at risk). Model 3 included model 2 plus high-density lipoprotein cholesterol, low-density lipoprotein cholesterol, high blood pressure, and *APOE*-ε4 genotype (14 495 patients at risk). Model 4 included model 3 plus diabetes, heart failure, coronary heart disease, myocardial infarction, and stroke (11 068 patients at risk).

^b^ *P* < .001.

^c^ *P* < .05.

Similarly, a sensitivity analysis removing dementia cases occurring less than 3 or more than 20 years from baseline or HWI produced consistent results (eTable 5 in Supplement 1). Among the exposed, 334 cases at a rate of 14.7 cases per 1000 person-years (95% CI, 13.7-15.8 cases per 1000 person-years) were observed, compared with 748 cases at a rate of 1.1 cases per 1000 person-years (95% CI, 1.0-1.2 cases per 1000 person-years) among the noninfected. Those who experienced an infection event had nearly 6 times the hazard of dementia after full adjustment (HR, 5.77; 95% CI, 4.92-6.76; *P* < .001).

### Subtypes of Infection and Dementia

Secondary analyses investigated the association between incident dementia and hospitalization with selected infection subtypes (Table 2). The rate of dementia diagnosis was significantly higher among those who experienced (vs not experienced) the following types of infection: respiratory, urinary tract, skin, blood and circulatory, and hospital acquired. After multivariable adjustment (Table 2), the top 3 infections associated with dementia were blood and circulatory system (HR, 2.13; 95% CI, 1.45-3.12; *P* < .001), urinary tract (HR, 1.98; 95% CI, 1.76-2.24; *P* < .001), and hospital acquired (HR, 1.96; 95% CI, 1.52-2.51; *P* < .001). Findings are consistent when defining HWI only with *ICD* position 1 (eTable 4 in Supplement 1). Only 64 patients had infections of the neurological system; therefore, these infections were not investigated as their own subtype.

### Subgroup Analyses of Any Hospitalization With Infection and Incidence of Dementia

**Figure 2.**
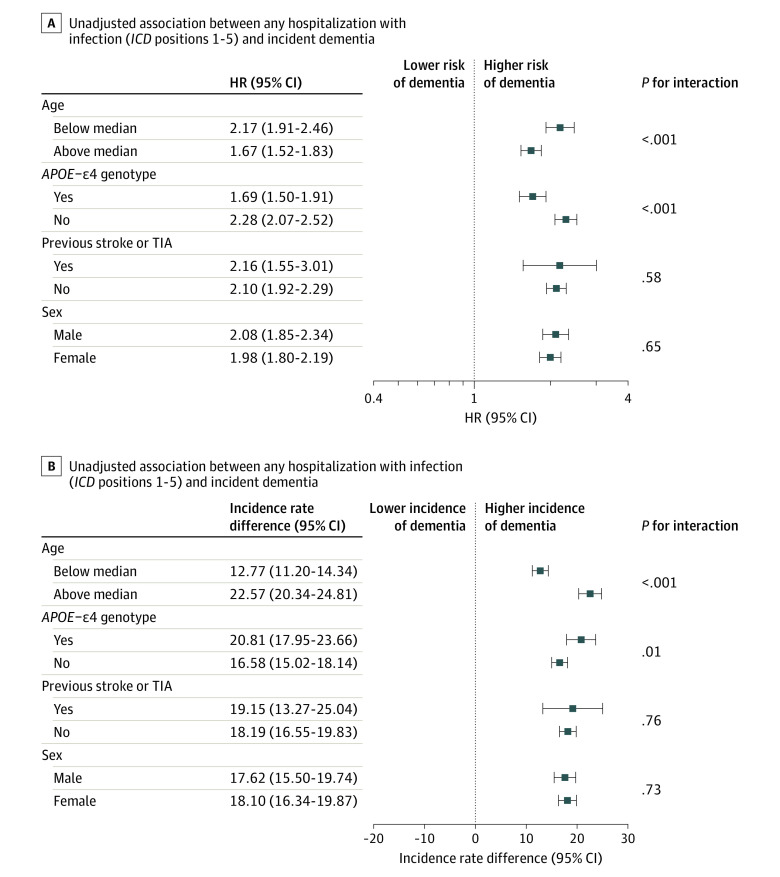
Unadjusted Association Between Any Hospitalization With Infection and Incident Dementia Data are shown for diagnoses with *International Classification of Diseases (ICD), Ninth Revision *or* Tenth Revision *positions 1-5 within substrata of age at baseline (median age, 54.45 years), *APOE*-ε4 genotype, previous stroke or transient ischemic attack (TIA), and sex in the multiplicative (A) and additive (B) scales. HR indicates hazard ratio.

Results across strata of key covariates are shown in Figure 2. HWI was a factor significantly associated with dementia diagnosis within all strata. There was evidence for statistical interaction between HWI and both *APOE*-ε4 genotype and age. Interestingly, measures of association on the additive scale were larger among the older age group and among *APOE*-ε4 carriers, whereas the reverse was true on the multiplicative scales; this is likely due to differences in the event rates between subgroups.

## Discussion

This cohort study investigated the association between HWI and incident dementia over 32 years of follow-up in the ARIC study. Participants with any HWI had an approximately 70% increased risk of dementia. Greater measures of association were found for urinary tract, blood and circulatory system, and hospital-acquired infections. Adjustments for age, sex, race, and education modestly attenuated the results, whereas further adjustment for *APOE*-ε4 genotype, behavioral, or vascular factors had limited influence.

These results were consistent, and even higher, in a sensitivity analysis excluding those who developed dementia less than 3 or more than 20 years after baseline or HWI. This analysis reduced the potential for reverse causality (ie, undiagnosed dementia leading to infection) by removing dementia cases identified within 3 years of HWI. In addition, dementia cases occurring 2 decades after the hospitalization event are possibly less plausibly linked.

There was evidence of interaction by age and *APOE*-ε4 genotype, but with opposite directions in multiplicative (submultiplicative) vs additive (superadditive) models. Consistent with our findings, a greater association between hospitalization and dementia among *APOE*-ε4 noncarriers (vs carriers) has been previously reported on the multiplicative scale^18^; however, results on the additive scale were not presented. Discordant findings by subgroups defined by high vs low levels of a known risk factor are plausible. This disagreement can arise because of large differences in absolute risk by the stratifying variable (eg, the small absolute risk among the unexposed individuals in the low-risk stratum inflates the relative risk in that same stratum). Interpretation of the additive models is generally more relevant for public health decision-making and interpretation of hypotheses regarding biological interactions. If confirmed, our findings suggest that the greatest population burden of dementia following HWI might be among those with advanced age or with *APOE*-ε4 risk alleles. Regardless, the consistent positive associations in all subgroups suggest that HWI is clinically relevant irrespective of age *APOE*-ε4 status.

These findings support and extend prior reports on the association between infection cognitive decline,^9^ brain imaging abnormalities,^10^ and dementia.^12^ Notably, our findings very closely replicated those of Sipilä et al,^12^ who found any HWI to be associated with a 60% increased dementia risk compared with our own observation of a 65% greater risk. This consistency is remarkable given the substantial variation in racial and ethnic composition, geographical region, and risk factor distributions between the study populations.

Higher risk of dementia has been previously linked to certain infection subtypes, including central nervous system infections,^19^ as well as common systemic infections,^20^ notably pneumonia.^21,22^ Our findings support the association between infection subtypes and dementia. It is biologically plausible that different infection subtypes could be associated with dementia. For instance, blood and circulatory system infections could impact dementia risk because of the brain’s high vascularity, whereas urinary tract infections could be more likely to lead to sepsis or bacteremia.^23^ In addition, there is substantial evidence that infections increase risk for vascular and metabolic illnesses known to be factors associated with increased risk of dementia, such as stroke, heart failure, coronary heart disease, and diabetes.^24,25,26,27^ The association between infections and increased cardiovascular disease risk has been previously demonstrated in the ARIC study.^28^ Finally, as suggested by the aforementioned studies, conditions associated with hospitalization itself are factors associated with the risk of cognitive decline and dementia, as shown by Eriksson et al^18^ in the Swedish Twin Registry studies.

The findings of the present study aid in explaining a share of previously unknown factors associated with risk of dementia. Further studies of this association and establishment of a causal relationship between infection and dementia are warranted, particularly through the investigation of the association between infections and known biomarkers of clinical or preclinical dementias or the inclusion of noninfection hospitalizations, frailty, or multicomorbidities. These investigations could create new avenues for dementia screening and prevention, such as incorporating questions about past infections into dementia screening efforts or enrollment criteria for prevention-oriented trials. In addition, movement toward lifelong care teams vs specialties working in isolation could potentially improve prevention and treatment efforts. These findings highlight the importance of infection prevention broadly and the need for identifying novel approaches to prevent deleterious effects of infections, including cognitive sequelae, throughout the lifetime.

### Limitations and Strengths

The present study has noteworthy limitations. The potential for residual or unmeasured confounding exists because of the nature of observational studies. Furthermore, some known dementia risk factors (eg, craniocerebral damage, confessional syndrome, and health care access), frailty, multicomorbidities, or other cognitive decline outcomes, including mild cognitive decline, were not available. Another limitation is potential loss to follow-up that could create selection biases, but, if the hypothesis is true, it is likely that loss to follow-up would bias toward the null, because those who experienced HWI (who are conceptually at higher risk of dementia) would be lost at higher rates than individuals without HWI presumably as a result of worse overall health status. Furthermore, in considering only infections that lead to hospitalization within the study time frame, the present study did not capture the burden of infections occurring before study initiation or infections that did not lead to a hospitalization. We also were not able to capture treatment approaches that could affect various aspects of one’s health, such as antibiotic use. Although presumably less impactful than infections that lead to hospitalization, chronic infections that do not result in hospitalization have been linked to dementia risk.^29^ In addition, we were not able to investigate infections of the neurological system on their own because of their small prevalence in this cohort (64 infections).

Despite these limitations, the present study has several strengths that advance prior research. First, we performed robust adjustments for several confounders, including genetic, behavioral, and vascular factors. Second, a long follow-up period of over 30 years increased our ability to capture both infections and incident dementia and better capture the time span of dementia development. The length and consistency of follow-up also aids in minimizing the risk of reverse causation, with the vast majority of dementia cases occurring more than 10 years after baseline. Third, the large sample size enabled additional analyses for several infection types and among important participant subgroups. Fourth, the ARIC study has established rigorous validated assessments for dementia, which includes several cognitive examinations, standardized diagnosis criteria, data sources with high reliability and accuracy, and comprehensive surveillance of HWI. These strengths enhance the validity of our exposure and outcome measures.

## Conclusions

This cohort study found that HWI was associated with higher risk of dementia over 32 years of follow-up in the ARIC study, a community-based, racially diverse sample of adults in the US. These findings bolster support for the hypothesis that infections contribute to the causes of dementia. Furthermore, they highlight that the risk of dementia differs by infection type, suggesting that specific pathophysiological aspects of an infection might affect dementia risk. Future evaluations should consider whether incorporating prior infection status into dementia screening could improve identification of preclinical disease stages. More importantly, our findings could inform approaches to mitigate or prevent dementias through accounting for the deleterious effects on cognitive and neurological health accrued throughout the lifetime due to infections and hospitalizations.

## Notes

Supplement 1. **eTable 1. ***International Classification of Diseases* (*ICD*) Codes Utilized for the Ascertainment of Hospitalization With Infection **eTable 2. **Cohort Characteristics by Any Hospitalization With Infection (*ICD* 1-5) During Follow-up, Censored at Dementia Diagnosis if Occurring in the Atherosclerosis Risk in Communities Study (1987-2019) **eTable 3. **Cumulative Incidence and Median Time to Any Hospitalization With Infection and to Subtypes of Hospitalization With Infection, Defined With *International Classification of Diseases *(*ICD*) Positions 1-5 and Position 1 Only, by Dementia Diagnosis **eTable 4. **Multivariable Adjusted Association Between Any Hospitalization With Infection and Infection Subtypes (*International Classification of Diseases, Ninth Revision *or *Tenth Revision*, Position 1) and Incident Dementia Among Participants in the Atherosclerosis Risk in Communities Study (1987-2019) **eTable 5. **Multivariable Adjusted Association Between Any Hospitalization With Infection (*International Classification of Diseases, Ninth Revision *or *Tenth Revision* Positions 1-5) and Incident Dementia Among Participants in The Atherosclerosis Risk in Communities Study (1987-2019), Censoring Dementia Cases Recorded Dementia Within 3 Years or Over 20 Years From First Hospitalization With Infection or Baseline, for Those Who Did Not Experience a Hospitalization With Infection

Supplement 2.
Data Sharing Statement
