# Supplementary material for: Incidence of Dementia Following Hospitalization With Infection Among Adults in the Atherosclerosis Risk in Communities (ARIC) Study Cohort
Source: JAMA Netw Open. 2023 Jan 9;6(1):e2250126. doi: 10.1001/jamanetworkopen.2022.50126 (PMC9857407; doi:10.1001/jamanetworkopen.2022.50126)
Supplement: Supplement 1. — eTable 1. International Classification of Diseases (ICD) Codes Utilized for the Ascertainment of Hospitalization With Infection eTable 2. Cohort Characteristics by Any Hospitalization With Infection (ICD 1-5) During Follow-up, Censored at Dementia Diagnosis if Occurring in the Atherosclerosis Risk in Communities Study (1987-2019) eTable 3. Cumulative Incidence and Median Time to Any Hospitalization With Infection and to Subtypes of Hospitalization With Infection, Defined With International Classification of Diseases (ICD) Positions 1-5 and Position 1 Only, by Dementia Diagnosis eTable 4. Multivariable Adjusted Association Between Any Hospitalization With Infection and Infection Subtypes (International Classification of Diseases, Ninth Revision or Tenth Revision, Position 1) and Incident Dementia Among Participants in the Atherosclerosis Risk in Communities Study (1987-2019) eTable 5. Multivariable Adjusted Association Between Any Hospitalization With Infection (International Classification of Diseases, Ninth Revision or Tenth Revision Positions 1-5) and Incident Dementia Among Participants in The Atherosclerosis Risk in Communities Study (1987-2019), Censoring Dementia Cases Recorded Dementia Within 3 Years or Over 20 Years From First Hospitalization With Infection or Baseline, for Those Who Did Not Experience a Hospitalization With Infection [file jamanetwopen-e2250126-s001.pdf]

Supplemental Online Content

Bohn B, Lutsey PL, Misialek JR, et al. Incidence of dementia following hospitalization with infection among adults in the Atherosclerosis Risk in Communities (ARIC) study cohort. *JAMA Netw Open*. 2023;6(1):e2250126. doi:10.1001/jamanetworkopen.2022.50126

eTable 1. *International Classification of Diseases* (*ICD*) Codes Utilized for the Ascertainment of Hospitalization With Infection
eTable 2. Cohort Characteristics by Any Hospitalization With Infection (*ICD* 1-5) During Follow-up, Censored at Dementia Diagnosis if Occurring in the Atherosclerosis Risk in Communities Study (1987-2019)
eTable 3. Cumulative Incidence and Median Time to Any Hospitalization With Infection and to Subtypes of Hospitalization With Infection, Defined With *International Classification of Diseases* (*ICD*) Positions 1-5 and Position 1 Only, by Dementia Diagnosis
eTable 4. Multivariable Adjusted Association Between Any Hospitalization With Infection and Infection Subtypes (*International Classification of Diseases, Ninth Revision* or *Tenth Revision*, Position 1) and Incident Dementia Among Participants in the Atherosclerosis Risk in Communities Study (1987-2019)
eTable 5. Multivariable Adjusted Association Between Any Hospitalization With Infection (*International Classification of Diseases, Ninth Revision* or *Tenth Revision* Positions 1-5) and Incident Dementia Among Participants in The Atherosclerosis Risk in Communities Study (1987-2019), Censoring Dementia Cases Recorded Dementia Within 3 Years or Over 20 Years From First Hospitalization With Infection or Baseline, for Those Who Did Not Experience a Hospitalization With Infection

This supplemental material has been provided by the authors to give readers additional information about their work.

© 2023 Bohn B et al. *JAMA Network Open.*

eTable 1: *International Classification of Diseases* (*ICD*) Codes Utilized for the Ascertainment of Hospitalization With Infection.
*Codes include further digits if existing (ex. J01 includes J01.1, J01.2, etc).

Infection Type	Ninth Revision	Tenth Revision
**Respiratory Infection**	460, 461, 462, 463, 464, 465, 466, 472, 473, 474.0, 475, 476.0, 476.1, 478.21, 478.22, 478.24, 478.29, 480, 481, 482, 483, 484, 485, 486, 487, 488, 490, 491.1, 494, 510, 511, 513.0, 518.6, 519.01	J00, J01, J02, J03, J04, J05, J06, J07, J08, J09, J11, J12, J13, J14, J15, J16, J17, J18, J20, J21, J31, J32, J36, J37, J35.1, J35.2, J35.3, J39.0, J39.1, J39.2, J40, J41.1, J47, J85.0, J85.1, J85.2, J86, J90, J91, J95.02, R09.1
**Urinary Tract Infection**	590, 595.0, 595.1, 595.2, 595.3, 595.4, 597, 598.0, 599.0	N10, N11, N12, N15.1, N15.9, N16, N28.84, N28.85, N28.86, N30.0, N30.1, N30.2, N30.3, N30.8, N34, N37, N39.0
**Digestive Tract Infection**	522.5, 522.7, 527.3, 528.3, 540, 541, 542, 566, 567, 569.5, 572.0, 572.1, 573.1, 573.2, 573.3, 575.0, 575.1	K04.0, K04.1, K04.4, K04.5, K04.6, K04.7, K11.3, K12.2, K35, K36, K37, K61, K63.0, K65, K67, K68.12, K68.19, K68.9, K71, K75.0, K75.1, K75.2, K75.3, K75.81, K75.89, K75.9, K77, K81
**Skin Infection**	680, 681, 682, 683, 684, 685, 686, 706.0	E82.2, K12.2, L01, L02, L03, L04, L05, L08, L70.2, L88, L98.0
**Blood/Circulatory System Infection**	390, 391, 392, 393, 421.0, 421.1, 422.0, 422.91, 422.92, 422.93, 790.7, 790.8	I00, I01, I02, I09.2, I33.0, I39, I40.0, I40.1, I40.8, I41, R78.81
**Hospital Acquired Infection**	996.6, 997.62, 998.5, 999.3	K68.11, T80.211, T80.212, T80.218, T80.219, T80.22, T80.29, T81.4, T82.6, T82.7, T83.5, T83.6, T84.5, T84.6, T84.7, T85.7, T87.4, T88.0
**Other Infections**	001-139, 254.1, 320, 321, 322, 323, 324, 325, 326, 331.81, 372.0, 372.1, 372.2, 372.3, 373.0, 373.1, 373.2, 382.0, 382.1, 382.2, 382.3, 382.4, 383, 386.33, 386.35, 388.60, 601, 604, 607.1, 607.2, 608.0, 608.4, 611.0, 614, 615, 616.0, 616.1, 616.3,	A01-A99, B01-B99, D86, E32.1, G00, G01, G02, G03, G04.00, G04.01, G04.02, G04.2, G04.30, G04.31, G04.32, G04.39, G04.81, G04.82, G04.83, G04.84, G04.85, G04.86, G04.87, G04.88, G04.89, G04.90, G04.91, G05, G06, G07, G08, G09, G92, G14,

© 2023 Bohn B et al. *JAMA Network Open.*

	616.4, 616.8, 670, 711, 730.0, 730.1, 730.2, 730.3, 730.8, 730.9	G93.7, H00, H01.0, H10, H32, H66.0, H66.1, H66.2, H66.3, H66.4, H67, H70, H83.0, H92.1, H95.0, H95.1, I32, K90.81, L44.4, L94.6, M60.009, M00, M01, M02.1, M35.2, M46.2, M46.3, N41, N45, N47.6, N48.1, N48.2, N49, N51, N61, N70, N71, N72, N73, N74, N75.1, N75.9, N76.0, N76.1, N76.2, N76.3, N76.4, N76.5, N76.81, N76.89, N77.1, O85, O86.12, O86.8,

© 2023 Bohn B et al. *JAMA Network Open.*

eTable 2: Cohort Characteristics by Any Hospitalization With Infection (*ICD* 1-5) During Follow-up, Censored at Dementia Diagnosis if Occurring in the Atherosclerosis Risk in Communities Study (1987-2019).

Characteristic	Patients, No. (%)			P value
	All (N = 15,688)	No Hospitalization with Infection (N = 9,689)	Any hospitalization with Infection (N = 5,999)	
**Demographics**				
Age at baseline, mean (SD)	54.7 (5.8)	54.3 (5.7)	55.2 (5.8)	<.001[a]
Age at or above the median (54.45)	7844 (50.0%)	4590 (47.4%)	3254 (54.2%)	<.001[b]
Sex				0.02[b]
Female	8658 (55.2%)	5275 (54.4%)	3383 (56.4%)	
Male	7030 (44.8%)	4414 (45.6%)	2616 (43.6%)	
Race				0.03[b]
Black	4210 (26.8%)	2659 (27.4%)	1551 (25.9%)	
White	11478 (73.2%)	7030 (72.6%)	4448 (74.1%)	
Race by center				<.001[b]
Black, MS	3728 (23.8%)	2361 (24.4%)	1367 (22.8%)	
Black, NC	482 (3.1%)	298 (3.1%)	184 (3.1%)	

© 2023 Bohn B et al. *JAMA Network Open.*

White, MD	3975 (25.3%)	2347 (24.2%)	1628 (27.1%)	
White, MN	3972 (25.3%)	2553 (26.3%)	1419 (23.7%)	
White, NC	3531 (22.5%)	2130 (22.0%)	1401 (23.4%)	
**Behaviors**				
Ever Drinker (Missing = 80)	11683 (74.9%)	7268 (75.4%)	4415 (74.0%)	0.06[b]
Ever Smoker (Missing = 11)	9152 (58.4%)	5481 (56.6%)	3671 (61.2%)	<.001[b]
Education (Missing = 26)				<.001[b]
Less than High School	3736 (23.9%)	2145 (22.2%)	1591 (26.6%)	
High School Degree (or equivalent)	6380 (40.7%)	3923 (40.6%)	2457 (41.0%)	
More than High School	5546 (35.4%)	3603 (37.3%)	1943 (32.4%)	
APOE-ε4 Genotype (Missing = 679)				0.003[b]
Negative	10379 (69.2%)	6340 (68.3%)	4039 (70.6%)	
Positive	4630 (30.8%)	2948 (31.7%)	1682 (29.4%)	
**Biomarkers at baseline**				

© 2023 Bohn B et al. *JAMA Network Open.*

Low-density lipoprotein cholesterol, mean (SD), mg/dL (Missing = 476)	137.7 (39.4)	137.8 (39.3)	137.6 (39.5)	<.001[a]
High-density lipoprotein cholesterol, mean (SD), mg/dL (Missing = 249)	51.6 (17.1)	52.2 (17.2)	50.5 (17.0)	<.001[a]
Triglycerides, median (IQR), mg/dL (Missing = 248)	110 [79-157]	107 [77-153]	114.5 [82-162]	<.001[c]
Estimated glomerular filtration rate serum creatinine, mean (SD), mL/min/1.73 m^2 (Missing = 149)	102.4 (15.9)	102.8 (15.4)	101.8 (16.6)	<.001[a]
**Comorbidities at baseline**				
High Blood Pressure (Missing = 80)	5471 (35.1%)	3181 (33.0%)	2290 (38.4%)	<.001[b]
Diabetes (Missing = 147)	1863 (12.0%)	950 (9.9%)	913 (15.3%)	<.001[b]
Atrial Fibrillation (Missing = 224)	37 (0.2%)	18 (0.2%)	19 (0.3%)	0.14 [b]
Stroke and transient ischemic attack (Missing = 3629)	732 (6.1%)	403 (5.4%)	329 (7.2%)	<.001[b]

© 2023 Bohn B et al. *JAMA Network Open.*

Heart Failure (Missing = 282)	746 (4.8%)	378 (4.0%)	368 (6.2%)	<.001[b]
Myocardial Infarction (Missing = 231)	650 (4.2%)	366 (3.8%)	284 (4.8%)	0.004[b]
Coronary Heart Disease (Missing = 339)	763 (5.0%)	417 (4.4%)	346 (5.9%)	<.001[b]

[a]P value was calculated with t test.

[b]P value was calculated with χ^2 or Fisher exact test, when appropriate.

[c]P value was calculated with Wilcoxon rank test.

© 2023 Bohn B et al. *JAMA Network Open.*

eTable 3: Cumulative Incidence and Median Time to Any Hospitalization With Infection and to Subtypes of Hospitalization With Infection, Defined With *International Classification of Diseases (ICD)* Positions 1-5 and Position 1 Only, by Dementia Diagnosis.

Exposure Construct	Dementia Diagnosis	Cumulative Incidence of Infection	Median [IQR] Time (Years) to Infection	Median [IQR] Time (Years) to Dementia Diagnosis Among Those with Infection
**Any Infection (ICD position 1-5)**	All	5999 (38.2%)	14.3 [7.3-21.8]	-
	No Dementia	4789 (37.7%)	13.9 [7.0-21.8]	-
	Dementia	1210 (40.7%)	15.3 [8.6-22.1]	25.6 [22.9-28.4]
**Any Infection (ICD position 1)**	All	3610 (23.0%)	15.6 [9.0-23.0]	-
	No Dementia	2868 (22.6%)	15.2 [8.6-23.0]	-
	Dementia	742 (24.9%)	16.8 [10.5-23.1]	25.7 [22.5-28.3]
**Respiratory Infection (ICD position 1-5)**	All	3083 (19.7%)	15.9 [9.2-23.1]	-
	No Dementia	2517 (19.8%)	15.6 [8.9-22.9]	-
	Dementia	566 (19.0%)	17.3 [10.4-23.5]	25.6 [22.7-28.4]
**Respiratory Infection (ICD position 1)**	All	1625 (10.4%)	15.2 [9.5-23.1]	-
	No Dementia	1311 (10.3%)	14.6 [9.2-22.9]	-
	Dementia	314 (10.6%)	16.8 [10.6-23.7]	25.4 [22.0-28.3]

© 2023 Bohn B et al. *JAMA Network Open.*

**Urinary Tract Infection (ICD position 1-5)**	All	1943 (12.4%)	17.7 [11.3-24.0]	-
	No Dementia	1470 (11.6%)	16.9 [10.5-23.8]	-
	Dementia	473 (15.9%)	19.7 [13.4-24.3]	25.7 [22.8-28.3]
**Urinary Tract Infection (ICD position 1)**	All	453 (2.9%)	18.4 [11.8-24.4]	-
	No Dementia	345 (2.7%)	17.4 [10/6-24.1]	-
	Dementia	108 (3.6%)	19.8 [15.3-25.5]	25.2 [22.5-28.3]
**Digestive Tract Infection (ICD position 1-5)**	All	581 (3.7%)	14.2 [7.4-21.2]	-
	No Dementia	460 (3.6%)	14.7 [8.5-21.9]	-
	Dementia	121 (4.1%)	12.9 [5.4-18.9]	26.1 [23.4-28.1]
**Digestive Tract Infection (ICD position 1)**	All	341 (2.2%)	13.2 [6.8-19.6]	-
	No Dementia	258 (2.0%)	13.5 [7.0-20.0]	-
	Dementia	83 (2.8%)	12.8 [5/4-18.6]	26.0 [23.3-28.2]
**Skin Infection (ICD position 1-5)**	All	919 (5.9%)	16.2 [9.8-22.8]	-
	No Dementia	721 (5.7%)	15.2 [8.6-22.6]	-
	Dementia	198 (6.7%)	19.5 [13.5-23.9]	25.9 [23.1-28.7]
**Skin Infection (ICD position 1)**	All	471 (3.0%)	16.7 [9.8-22.8]	-
	No Dementia	374 (2.9%)	15.7 [8.3-22.7]	-

© 2023 Bohn B et al. *JAMA Network Open.*

	Dementia	97 (3.3%)	19.7 [14.4-22.9]	26.1 [23.1-28.9]
**Blood/Circulatory System Infection (ICD position 1-5)**	All	191 (1.2%)	17.6 [12.6-23.1]	-
	No Dementia	151 (1.2%)	17.4 [12.8-22.8]	-
	Dementia	40 (1.3%)	19.5 [14.4-22.9]	24.9 [23.1-28.9]
**Blood/Circulatory System Infection (ICD position 1)**	All	58 (0.4%)	15.4 [10.6-21.2]	-
	No Dementia	43 (0.3%)	14.6 [9.5-20.7]	-
	Dementia	15 (0.5%)	17.1 [12.8-22.6]	23.1 [17.9-28.0]
**Hospital Acquired Infection (ICD position 1-5)**	All	499 (3.2%)	14.4 [7.9-20.6]	-
	No Dementia	302 (3.2%)	14.3 [7.8-20.5]	-
	Dementia	97 (3.3%)	14.5 [9.1-21.4]	25.7 [22.1-28.9]
**Hospital Acquired Infection (ICD position 1)**	All	353 (2.3%)	15.4 [10.1-21.8]	-
	No Dementia	284 (2.2%)	15.0 [9.8-21.4]	-
	Dementia	69 (2.3%)	16.3 [11.6-22.9]	25.2 [22.1-28.8]

© 2023 Bohn B et al. *JAMA Network Open.*

eTable 4: Multivariable Adjusted Association Between Any Hospitalization With Infection and Infection Subtypes (*International Classification of Diseases, Ninth Revision* or *Tenth Revision*, Position 1) and Incident Dementia Among Participants in the Atherosclerosis Risk in Communities Study (1987-2019)

Exposure Construct and Infection Status	Patients, No.		Rate, No. of cases per 1000 person-years (95% CI)	HR (95% CI)[a]				
	Total (N = 15688)	With Dementia		Model 0	Model 1	Model 2	Model 3	Model 4
**Any Infection**								
Yes	3610	742	26.7 (24.9-28.7)	1.95 (1.8-2.13)[b]	1.64 (1.5-1.78)[b]	1.63 (1.5-1.78)[b]	1.59 (1.46-1.74)[b]	1.65 (1.49-1.83)[b]
No	12078	2233	6.7 (6.4-7)	1 [Reference]	1 [Reference]	1 [Reference]	1 [Reference]	1 [Reference]
**Respiratory Infection**								
Yes	1625	314	28.4 (25.5-31.7)	2.03 (1.8-2.28)[b]	1.59 (1.41-1.79)[b]	1.57 (1.39-1.76)[b]	1.55 (1.37-1.75)[b]	1.57 (1.36-1.82)[b]
No	14063	2661	7.6 (7.3-7.9)	1 [Reference]	1 [Reference]	1 [Reference]	1 [Reference]	1 [Reference]
**Urinary Tract Infection**								

© 2023 Bohn B et al. *JAMA Network Open.*

Yes	453	108	37.4 (31.1-45)	2.2 (1.81-2.67)[b]	1.77 (1.46-2.15)[b]	1.82 (1.5-2.2)[b]	1.76 (1.44-2.16)[b]	1.97 (1.56-2.48)[b]
No	15235	2867	8 (7.7-8.3)	1 [Reference]	1 [Reference]	1 [Reference]	1 [Reference]	1 [Reference]
**Digestive Tract Infection**								
Yes	341	83	21.2 (17.1-26.2)	1.34 (1.08-1.67)[c]	1.27 (1.02-1.58)[d]	1.25 (1-1.56)	1.21 (0.96-1.52)	1 (0.75-1.32)
No	15347	2892	8.1 (7.8-8.4)	1 [Reference]	1 [Reference]	1 [Reference]	1 [Reference]	1 [Reference]
**Skin Infection**								
Yes	471	97	27.4 (22.5-33.3)	1.67 (1.36-2.04)[b]	1.49 (1.21-1.82)[c]	1.51 (1.23-1.85)[b]	1.51 (1.22-1.86)[c]	1.58 (1.23-2.02)[c]
No	15217	2878	8 (7.8-8.3)	1 [Reference]	1 [Reference]	1 [Reference]	1 [Reference]	1 [Reference]
**Blood/Circulatory System Infection**								
Yes	58	15	40.5 (24.7-66.4)	2.99 (1.8-4.96)[b]	3.71 (2.23-6.17)[b]	3.68 (2.22-6.12)[b]	3.49 (2.06-5.91)[b]	3.46 (1.86-6.47)[b]
No	15630	2960	8.2 (7.9-8.5)	1 [Reference]	1 [Reference]	1 [Reference]	1 [Reference]	1 [Reference]

© 2023 Bohn B et al. *JAMA Network Open.*

**Hospital Acquired Infection**								
Yes	353	69	29.3 (23.2-36.9)	1.88 (1.48-2.39)[b]	1.88 (1.48-2.39)[b]	1.86 (1.46-2.37)[b]	1.81 (1.4-2.34)[b]	2.03 (1.51-2.71)[b]
No	15335	2906	8.1 (7.8-8.4)	1 [Reference]	1 [Reference]	1 [Reference]	1 [Reference]	1 [Reference]

Abbreviation: HR, Hazard Ratio

[a]HRs were derived from Cox proportional hazards models: Model 0 was unadjusted (15688 participants at risk). Model 1 was adjusted for age, sex, race by center, and education (15662 participants at risk). Model 2 included model 1 plus smoking and drinking (15581 participants at risk). Model 3 included model 2 plus high-density lipoprotein cholesterol, low-density lipoprotein cholesterol, high blood pressure, and APOE-ε4 genotype (14495 participants at risk). Model 4 included model 3 plus diabetes, heart failure, coronary heart disease, myocardial infarction and stroke (11068 participants at risk).

[b]$P < .001$

[c]$P < .01$

[d]$P < .05$

© 2023 Bohn B et al. *JAMA Network Open.*

eTable 5: Multivariable Adjusted Association Between Any Hospitalization With Infection (*International Classification of Diseases, Ninth Revision* or *Tenth Revision* Positions 1-5) and Incident Dementia Among Participants in the Atherosclerosis Risk in Communities Study (1987-2019), Censoring Dementia Cases Recorded Dementia Within 3 Years or Over 20 Years From First Hospitalization With Infection or Baseline, for Those Who Did Not Experience a Hospitalization With Infection.

Exposure Construct and Infection Status	Patients, No.		Rate, No. of cases per 1000 person-years (95% CI)	HR (95% CI)[a]				
	Total (N = 15688)	With Dementia		Model 0	Total (N = 15688)	With Dementia	Model 3	Model 4
**Any Infection**								
Yes	5999	334	14.7 (13.7-15.8)	7.78 (6.82-8.88)[b]	6.07 (5.31-6.94)[b]	6.06 (5.3-6.93)[b]	5.82 (5.06-6.70)[b]	5.77 (4.92-6.76)[b]
No	9689	748	1.1 (1.0-1.2)	1 [Reference]	1 [Reference]	1 [Reference]	1 [Reference]	1 [Reference]

Abbreviation: HR, Hazard Ratio

[a]HRs were derived from Cox proportional hazards models: Model 0 was unadjusted (15688 participants at risk). Model 1 was adjusted for age, sex, race by center, and education (15662 participants at risk). Model 2 included model 1 plus smoking and drinking (15581 participants at risk). Model 3 included model 2 plus high-density lipoprotein cholesterol, low-density lipoprotein cholesterol, high blood pressure, and APOE-ε4 genotype (14495 participants at risk). Model 4 included model 3 plus diabetes, heart failure, coronary heart disease, myocardial infarction and stroke (11068 participants at risk).

[b]$P < .001$

© 2023 Bohn B et al. *JAMA Network Open.*
