# Supplementary material for: Incidence of Dementia Following Hospitalization With Infection Among Adults in the Atherosclerosis Risk in Communities (ARIC) Study Cohort
Source: JAMA Netw Open. 2023 Jan 9;6(1):e2250126. doi: 10.1001/jamanetworkopen.2022.50126 (PMC9857407; doi:10.1001/jamanetworkopen.2022.50126)
Supplement: Supplement 2. — Data Sharing Statement [file jamanetwopen-e2250126-s002.pdf]

Data Sharing Statement

Bohn. Incidence of Dementia Following Hospitalization With Infection Among Adults in the Atherosclerosis Risk in Communities (ARIC) Study Cohort. *JAMA Netw Open*. Published January 09, 2023. doi:10.1001/jamanetworkopen.2022.50126

Data
Data available: Yes
Data types: Deidentified participant data
How to access data: https://biolincc.nhlbi.nih.gov/studies/aric/
When available: With publication

Supporting Documents
Document types: None

Additional Information
Who can access the data: Data will be made available upon reasonable request.
Types of analyses: Data will be made available upon reasonable request.
Mechanisms of data availability: Data will be made available upon reasonable request.
